# Supplementary material for: Predicting visual function by interpreting a neuronal wiring diagram
Source: Nature. 2024 Oct 2;634(8032):113–23. doi: 10.1038/s41586-024-07953-5 (PMC11446822; doi:10.1038/s41586-024-07953-5)
Supplement: Supplementary file 6 — CRF and ERF predictions for individual TmY4 and TmY9 cells. Analogous to Supplementary Data 3, but for TmY target types. Shown are the top four monosynaptic pathways, the strongest pathway passing through each of the top ten intermediary types (ranking from Extended Data Fig. 7), and the trisynaptic pathway Tm1–TmY–Dm3–TmY (see the section entitled Prediction of spatial normalization). [file 41586_2024_7953_MOESM6_ESM.zip › DataS4/TmY9q/720575940635448824.pdf]

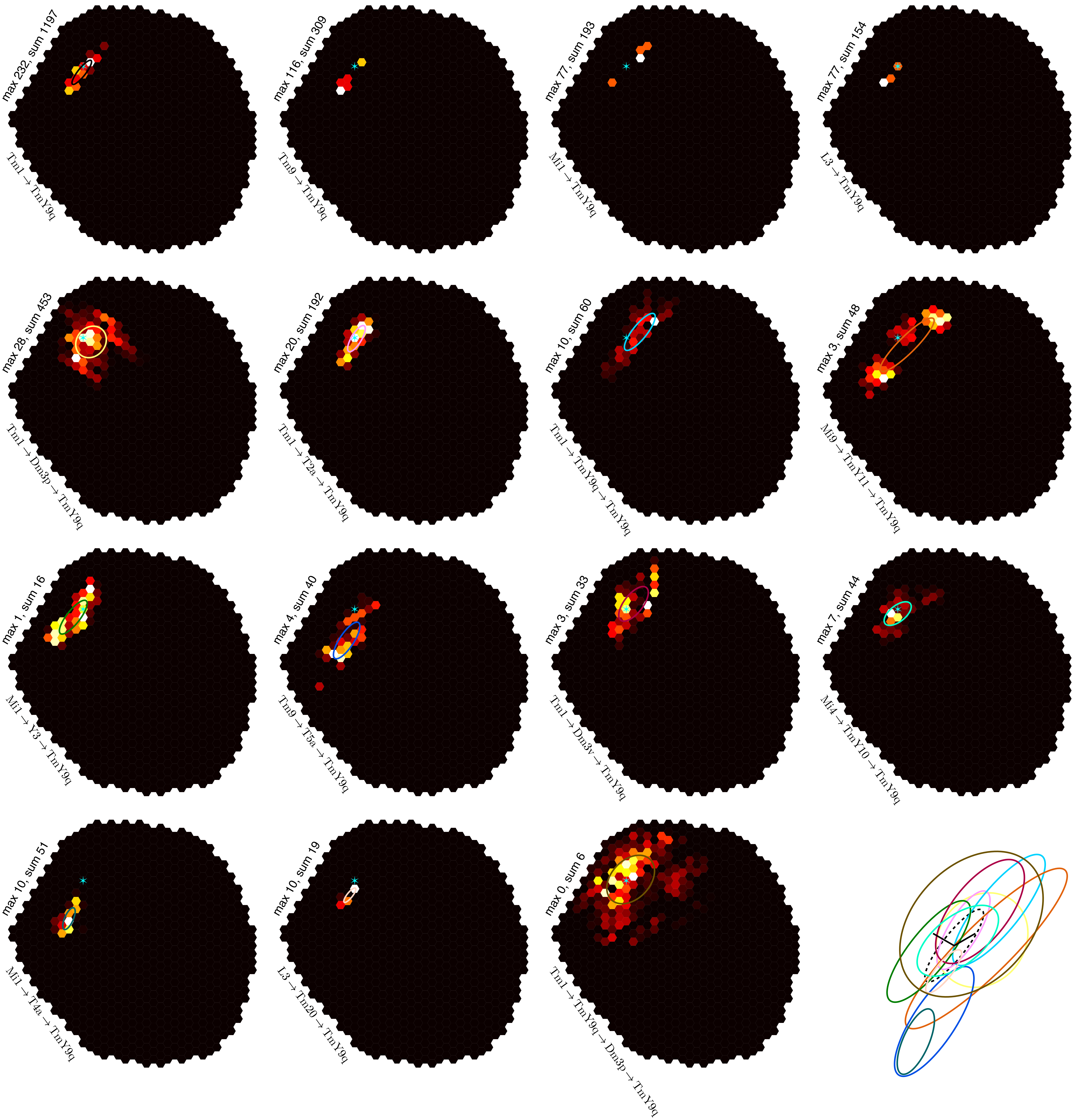
max 232, sum 1197
Tm1 → TmY9q
max 116, sum 309
Tm9 → TmY9q
max 77, sum 193
Mi1 → TmY9q
max 77, sum 154
L3 → TmY9q
max 28, sum 453
Tm1 → Dm3p → TmY9q
max 20, sum 192
Tm1 → T2a → TmY9q
max 10, sum 60
Tm1 → TmY9q → TmY9q
max 3, sum 48
Mi9 → TmY11 → TmY9q
max 1, sum 16
Mi1 → Y3 → TmY9q
max 4, sum 40
Tm9 → T5a → TmY9q
max 3, sum 33
Tm1 → Dm3v → TmY9q
max 7, sum 44
Mi4 → TmY10 → TmY9q
max 10, sum 51
Mi1 → T4a → TmY9q
max 10, sum 19
L3 → Tm20 → TmY9q
max 0, sum 6
Tm1 → TmY9q → Dm3p → TmY9q
